# Nanomaterials from Textile Waste for Purification and Environmental Applications

**DOI:** 10.3390/polym17233098

**Published:** 2025-11-21

**Authors:** Niyi Gideon Olaiya, Md. Al-Amin, Kaifur Rashed, Chrysanthos Maraveas

**Affiliations:** 1 Department of Plastics Engineering, University of Massachusetts Lowell, 1 University Ave., Lowell, MA 01854, USA; md_alamin@student.uml.edu (M.A.-A.); kaifur_rashed@uml.edu (K.R.); 2 Department of Industrial and Production Engineering, Federal University of Technology, Akure 340110, Nigeria; 3 Department of Natural Resources and Agricultural Engineering, Agricultural University of Athens, 11855 Athina, Greece

**Keywords:** waste, textile, sustainability, environmental, recycling

## Abstract

The growing scarcity of natural renewable resources has accelerated interest in producing nanomaterials from waste streams. Nanomaterials offer exceptional reinforcement capabilities for advanced composites, driving the need for sustainable and scalable production routes. While prior reviews have broadly examined nanomaterial synthesis from biomass or industrial residues, they often overlook textile waste as a strategic feedstock. This review uniquely focuses on the upcycling of textile waste—one of the most abundant yet underutilized waste streams—into high-value nanomaterials, thereby advancing circular economy principles. Unlike earlier studies that primarily discuss energy recovery or generic recycling, this work systematically explores mechanical, chemical, and thermal conversion routes tailored for textiles, leading to the production of cellulose nanofibers, cellulose nanocrystals, and carbon nanoparticles, which represent a significant class of biodegradable nanomaterials. Furthermore, a comprehensive analysis of the physicochemical properties of the nanomaterials and their emerging applications in water purification and environmental remediation is provided. An alternative pathway for nanomaterial synthesis from waste rather than renewable sources, providing information on the effective extraction of nanomaterials from mixed fiber compositions and dye residues present in textile waste, is also highlighted. By addressing current challenges and outlining future research directions, this review establishes a roadmap for sustainable textile waste valorization, marking a critical step toward eco-friendly nanomaterial production.

## 1. Introduction

The demand for composite development for high-strength applications has generated research interest in nanomaterial development from waste sources [1]. Nanomaterials, as opposed to micro-sized materials, have a unique reinforcement ability to produce a composite with unique strength because of their surface area and are used for several applications [2,3]. Nanomaterials are fundamental for reinforcing polymers to improve properties, such as strength, electrical conductivity, and thermal stability, among others. Demands for major nanomaterials like nanocellulose and carbon nanoparticles have increased significantly in recent decades. The production of biodegradable nanomaterials from non-renewable resources has caused severe depletion of the environment. This has spurred the utilization of alternative sources to manufacture biodegradable nanomaterials, such as textile waste.

The growing accumulation of textile waste poses a significant environmental challenge, driven by the rapid expansion of global textile production and consumption [4,5]. The United Nations (UN) forecasts that by 2030, global textile waste will reach 134 million tons, increasing by approximately 92 million tons annually [6]. Sadly, while 87% of the waste ends up in landfills or is incinerated, only 13% is recycled, with a mere 1% transformed into new textiles [7]. Textile waste upcycling has been proposed to be a major way to combat these issues, and various technologies are under development to turn this waste into useful products [8]. Based on UN statistics, the growing volume of textile waste, coupled with very low recycling rates, suggests possible underdevelopment in the upcycling and recycling process. Therefore, the use of textile waste as a source of nanomaterial is a novel strategy not just in waste upcycling but in new material development. However, manufacturing nanomaterials from recycled textile waste poses significant challenges. Current disposal methods for textile waste, such as incineration, landfilling, and scattering, reveal that the search for self-biodegradable alternatives to plastic packaging and textile products remains in its early stages [9]. Textile waste features a wide range of fused polymer fiber structures that are interconnected by various physical and chemical bonds, making separation difficult. While some studies have explored recycling cotton, rayon, and jute waste to extract cellulose for nanofiber production, large-scale cellulose recovery still relies predominantly on natural wood fibers. A critical barrier is the requirement for extensive pretreatment to remove dyes and chemical additives before cellulose extraction and subsequent conversion into functionalized nanocellulose [4].

Recycling textile waste can provide significant cost benefits in the synthesis of sustainable nanomaterials [10]. Analysis of case studies indicates that the costs associated with recycling, pretreatment, and valorization can be comparable to virgin feedstocks, and that lower material and energy overheads can readily offset the economic disadvantage imposed by diminished supply reliability [11,12]. Techno-economic analyses project production costs of approximately $3–4/kg for cellulose nanocrystals (CNCs). These estimates are far below current market prices, where CNC often sells for more than $50/kg, although reliance on ionic liquids and expensive enzymes can significantly increase overall cost [13]. Mechanical recycling methods, such as shredding and milling, are considered cost-effective but often require integration with chemical treatments to achieve high-quality outputs. Scaling up and advancing processing technologies could further reduce costs, enabling circular economy models that recover high-value end-of-life materials and minimize cradle-to-grave impacts [14].

A variety of pathways exist to convert textile fibers into nanomaterials, each with its own advantages and limitations. Mechanical methods, such as grinding, milling, and high-intensity ultrasonication, are simple and avoid harsh chemicals, making them relatively inexpensive [15]. However, purely mechanical treatment tends to shorten fibers and consume high energy and often yields lower-crystallinity nanofibers unless combined with chemical steps [16]. Chemical treatments, such as acid hydrolysis or TEMPO-mediated oxidation, can produce high-aspect-ratio nanocellulose with precise surface chemistry. For example, combining sulfuric acid hydrolysis with sonication yielded CNCs from denim or cotton waste with widths of 10–12 nm. Other chemical schemes, including alkali pretreatment followed by controlled oxidation, have been reported; these typically involve multiple stages, such as alkaline washing and bleaching to remove non-cellulose components and then acid or oxidant treatment to liberate nanocellulose [17,18]. Biological methods, such as enzymatic hydrolysis or microbial pretreatment operated under mild conditions, can yield very pure nanocellulose, but they rely on expensive enzymes or long fermentation times that are challenging to scale. Consequently, hybrid strategies combining mechanical and chemical steps are often employed to balance cost, quality, and environmental performance [19].

Nanomaterials produced from textile waste must meet specific performance benchmarks to achieve comparable functionalities to conventional nanomaterials derived from traditional feedstocks. Key indicators include crystallinity, purity, particle size distribution, specific surface area, catalytic activity, optical properties, and stability [9]. Comparative analysis with market-available materials synthesized from conventional sources highlights the ability of waste-derived pathways to produce materials with similar specifications. While findings indicate that performance parity is feasible, challenges remain in ensuring reproducibility, reliability, and scalable production [20]. Information on purification methods, alongside bottlenecks at available routes, is studied to delineate production stages where impurity removal is integral [21,22,23]. Processing and performance considerations, however, significantly differentiate pathways of interest and pose additional trade-offs.

Although research has explored converting textile waste into bio-oils and fuels, its valorization into nanomaterials is comparatively underdeveloped [24]. Existing literature lacks a unified framework linking synthesis routes, physicochemical properties, and environmental performance. Specifically, the effects of mixed-fiber composition, dye residues, and chemical treatments on nanomaterial quality and application potential remain insufficiently addressed. This review aims to bridge these gaps by providing a systematic analysis of textile-waste-derived nanomaterials, their synthesis pathways, and emerging applications in sustainable material development and environmental remediation. By identifying key challenges and opportunities, this work seeks to advance the integration of textile waste recycling into the broader context of the circular economy and nanotechnology innovation. This review only highlights the direct conversion of textile waste into nanocellulose and nanoparticles. The recycling method highlighted here cannot be used for general waste recycling.

## 2. Methodology

The present review employed a comprehensive approach to collect, analyze, and synthesize scientific information related to the conversion of textile waste into functional nanomaterials. Relevant literature was gathered from major databases, including Scopus, Web of Science, ScienceDirect, and Google Scholar, covering the period between 2010 and 2025. The search was performed using a combination of keywords such as “textile waste recycling”, “nanocellulose from textile waste”, “recycled textile nanoparticles”, “hydrothermal carbonization”, “mechanical recycling of fabrics”, and “environmental applications of nanomaterials”. Only peer-reviewed journal articles, book chapters, and review papers written in English were included. Studies were selected based on their relevance to the preparation, characterization, and application of nanomaterials derived from textile waste, including nanocellulose, nanofibers, and carbon nanoparticles. Articles focusing exclusively on energy recovery, non-nanomaterial recycling, or lacking sufficient methodological detail were excluded. Data extraction focused on three major domains: processing techniques, material characteristics, and functional performance. The studies were analyzed to compare different recycling routes, including mechanical, chemical, and thermal, to evaluate their efficiency, scalability, and environmental impact. Particular attention was given to correlating the processing parameters with the physicochemical properties of the nanomaterials, such as morphology, yield, crystallinity, and surface chemistry performance in applications like filtration, adsorption, and environmental remediation. This methodological framework provided a comprehensive basis for evaluating current progress, identifying limitations, and suggesting future directions for the sustainable upcycling of textile waste into multifunctional nanomaterials.

## 3. Recycling of Textile Waste

**Figure 1:**
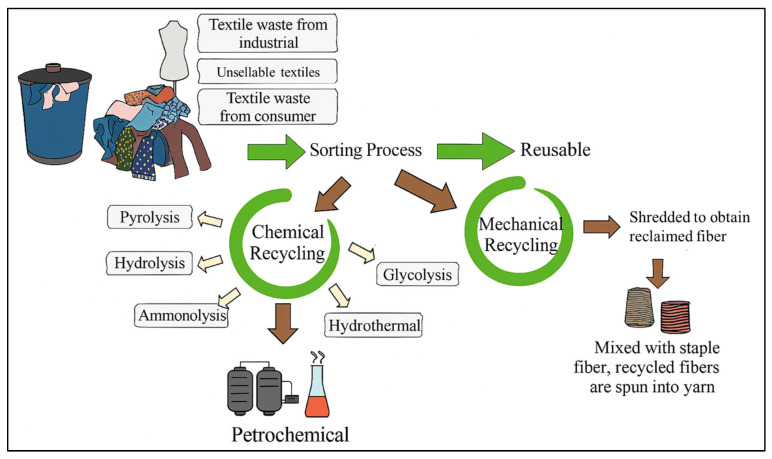
Potential methods of textile recycling [25].

Several methods have been used to recycle textile waste with minimum environmental impact. Figure 1 shows a schematic diagram of textile waste recycling techniques. All recycling techniques start with sorting. Usually, the waste materials are sorted based on the fiber content to maximize recycling output. Textile waste often consists of cotton, wool, silk, and synthetic fibers, and the effectiveness of the sorting process affects the nanomaterial output from the techniques. The sorted textile waste is washed to remove contaminants, such as dirt and grease, among others, before recycling through the following methodologies.

### 3.1. Mechanical Recycling

Mechanical recycling of textiles is the technique of turning textile fabric back into fibers without the use of chemical agents [26]. This method presents a viable solution to mitigate the environmental burdens associated with both production and consumption, as it facilitates the generation of new fibers from waste textiles that have comparable quality to those produced during initial manufacturing, in both polyester and cotton [27]. Nonetheless, optimization of this process is critical due to the high contamination levels present in waste textiles, which serve as feedstock for the recycling processes. Currently, the principal processes used in textile recycling involve energy recovery and downcycling to lower-grade products when compared to primary production [28]. These methods are becoming increasingly seen as being out of date and seriously harming the environment, especially when it comes to carbon dioxide (CO_2_) emissions. On the other hand, compared to traditional primary production processes, material recycling or upcycling techniques can reduce resource utilization [29].

**Figure 2:**
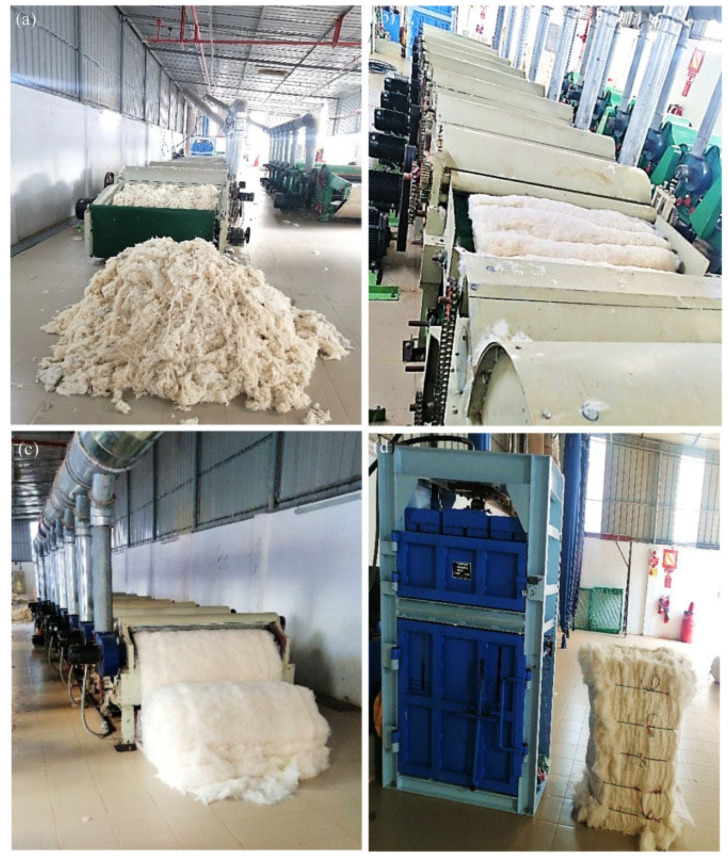
Mechanical recycling involves: (**a**) feeding material into the shredding machine, (**b**) shredding across multiple zones, (**c**) delivering material post-shredding, and (**d**) creating the final bale making [31].

A methodical series of steps, including sorting, cutting, shredding, and fiber separation, is involved in the mechanical processing of textile waste [30]. Textile waste is mechanically processed, as seen in Figure 2. The waste textiles are clipped and shredded until they are fibrous. A cutting guillotine machine is employed, featuring a sharp blade equipped with an in-built sharpening mechanism that accurately cuts designated fabric into pieces measuring 1 inch by 1 inch. Subsequently, the fabric is conditioned by applying a 2% moisture content relative to the fabric’s weight for a minimum duration of 12 h to reduce the risk of fire during the shredding process. This precaution is essential [31]. The clipped cloth fragments are then further reduced in size to 5 mm or less using an industrial shredding machine.

The machine in question comprises six carding cylinders, each outfitted with opposing sets of robust and sharp wires [31]. These wires, which can be fixed or floating, are used to hold the fibers in place in flat carding machines. The material must go through multiple sequential procedures to be carded. The first step is to turn the fibrous material from a coarsely jumbled mixture into a rough web of the shortest straightened strands. This web is then compressed into a continuous thread, increasing its uniformity in size and removing dirt and motes simultaneously [31]. Ultimately, these strands are converted into individual fibers by applying substantial and coarse carding actions with the sharp wires of the carding cylinders. The extracted fibers are then packaged into bales, which are later transported to the mixing room to form various blends, including recyclables, dyed and non-dyed cotton, and polyester–cotton blends in specified proportions.

### 3.2. Chemical Recycling

Chemical recycling is one of the many waste management techniques that has shown great promise in reducing the harmful environmental effects of textile waste [25]. Chemical recycling occurs when the textile waste is depolymerized into monomers or polymerized to make new fibers. The chemical recycling of textile waste, such as cotton, uses the dissolution approach with organic solvents and ionic liquids to dissolve cellulose polymers [27]. Compared with mechanical recycling, which can result in a reduction in fiber quality and integrity, chemical recycling preserves the quality of the fiber similar to the virgin material [25].

Chemical recycling has more advantages than other recycling methods because it can be used for various types of textile waste. The chemical process can be adapted to any mixture or source of textile waste without significant complexity [32]. This means that chemical recycling for textile waste containing contaminants such as dyes, colorations, and polyester can easily be separated from the cellulose, which is the main ingredient needed—without rigorous pressure or energy waste—which is usually the challenge with mechanical recycling [33]. Furthermore, chemical recycling effectively separates textile waste from hazardous industrial material without human contact with those contaminants.

Several chemical technology processes have been implemented or developed for chemical recycling. Such processes include hydrolysis, solvolysis, and depolymerization, to mention a few [32]. Chemical recycling procedures aim to reduce complex chemicals to their monomers for subsequent reuse. This process is often called depolymerization, and it involves hydrolysis using alkaline (sodium hydroxide (NaOH) or acidic (such as sulfuric acid) solutions. Hydrolysis involves cationic substitutions at high temperature [34]. Textile waste is soaked in the solution, which enhances swelling and dissolution of all contaminants from the waste. Employing this technique allows for the recovery of decomposed polyester powders, enabling their subsequent reuse.

Cation-exchange resin under acidic conditions can facilitate the recycling of polyester resins [35]; however, it involves using hazardous chemicals that require prior treatment for separation during the initial stage of the recycling process. Commercial cation-exchange resins are crosslinked macroporous polymer beads bearing sulfonic acid groups (–SO_3_H). The –SO_3_^−^·H^+^ pair provides Brønsted acidity and an ion-exchange site that can release H^+^ or exchange that H^+^ for cations, such as Na^+^, Cu^2+^, and dye cations [36]. The sulfonic acid groups act as heterogeneous Brønsted acid catalysts. They protonate labile bonds, such as ester bonds in PET, glycosidic bonds in cellulose, and solvated oligomers by lowering the activation energy for depolymerization. Effectiveness depends on substrate accessibility, solvent, and resin acidity/crosslinking [37]. In contrast, the hydrolysis of polyester in NaOH solution is considerably more economical and straightforward than the method involving a cation-exchanged resin [38]. Nevertheless, it necessitates reaching a high temperature of 80 °C, which limits batch processing efficiency. While cotton itself is insoluble in NaOH solution, the dye present on the fabric is readily eliminated [39]. Complete separation of dyed cotton can be accomplished by treating the material with approximately 2% NaOH solution for one hour [40].

Solvolysis is a type of chemical recycling process for polyester and its blends, as it can break down fabrics into their constituent monomers for reuse. This method is an alternative to mechanical recycling and is specifically used for complex textile blends that are difficult to recycle mechanically. Solvolysis leads to the recovery of monomers at low to moderate temperatures [41]. It is a chemical depolymerization process wherein a polymer chain is cleaved by reaction with a solvent or reagent under elevated temperature and pressure in the presence of a catalyst. For example, in the case of PET, ester bonds are hydrolyzed, glycolyzed, or methanolized. The solvent, including water, glycol, and methanol, attacks the carbonyl carbon of the ester linkage. Then it forms a tetrahedral intermediate, which then collapses by releasing the glycol backbone fragment and yielding the monomer or oligomer, such as bis-hydroxyethyl terephthalate. This reaction restores monomeric feedstocks, which can be purified and repolymerized [42].

This screw-based solvolysis approach is the most effective chemical recycling technique for synthetic polycondensation polymers [43]. Achieving high plastic purity is crucial for mechanical and solvolysis, necessitating meticulous sorting of the textiles before recycling. Typically, a high monomer recovery solvolysis process is supported by low temperatures and high pressures [34]. This method shows promise for recycling PET fibers, yielding high-quality monomers without producing any toxic by-products [44].

**Figure 3:**
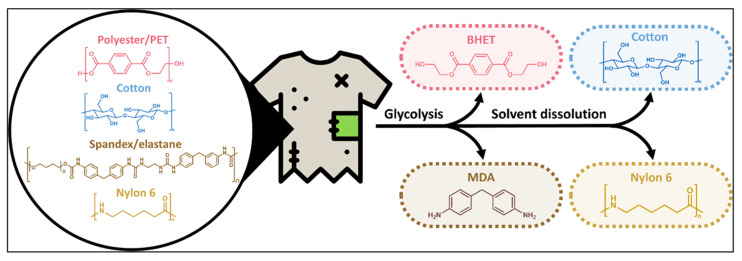
Overview of the chemical full recycling process using glycolysis and solvolysis [46].

Glycolysis, or the hydrolysis of the polymer, currently represents the standard industrial methodology for recycling [45]. Glycol first permeates the polymer, causing it to swell; the absorbed glycol subsequently interacts with an ester bond in the polymer chain. This swelling accelerates both diffusion and glycolysis reaction rates. Notably, the reaction rate is enhanced by the polymer’s surface area [34]. Andini et al. [46] demonstrated the chemical conversion of postconsumer mixed textile waste using microwave-assisted glycolysis over a ZnO catalyst, followed by solvent dissolution (Figure 3). Using the catalyst and process heat allows for the rapid depolymerization of polyester and spandex to their monomers within 15 min. Following depolymerization, a straightforward solvent dissolution technique separates cotton and nylon fibers. This process ensures the complete degradation of polyester and spandex into their monomeric forms, while preserving the integrity of the cotton and nylon fibers throughout the procedure. Their result describes the potential for sustainable recycling and provides a techno-economic analysis of the economic feasibility of the process.

Kuma et al. analyzed the hydrolysis of old PET/jute fabric, employing a precise composition of p-toluene sulfonic acid and water, which yields high-quality monomers and oligomers suitable for reuse within the plastic, resin, and fiber industries [47]. The dissolution and co-hydrolysis processes were scrutinized through Fourier-transform infrared spectroscopy (FTIR), 1H–15N heteronuclear multiple quantum coherence nuclear magnetic resonance (HMQC NMR), and 1H relaxometry measurements, thereby enhancing the comprehension of the co-hydrolysis mechanisms of PET and jute fabrics at the molecular level [48]. Furthermore, there is an imperative need for simple and efficient recycling methodologies for cotton/polyester textile blends. Characterization of cotton and polyester fibers, both pre- and post-hydrolysis, was conducted by Mu et al., utilizing various analytical and imaging techniques, focusing on size and morphology [49]. Additionally, the chemical structure of recycled polyester was characterized. The recovery of cotton from cotton/polyester textile blends utilizing a recyclable ionic liquid and metal salt additives presents a feasible approach and offers potential to produce new textile fabrics within a closed-loop recycling system.

The strength of recycled fibers is contingent upon the source of the textiles, the recycling methods employed, and the specific nature of the fibers [50]. Even though a significant amount of polyester (PET/PETG) plastic can be recycled, the procedures involved in recovering polyester fibers provide persistent technical difficulties. Occasionally, recycling procedures that combine mechanical and chemical elements are used to maximize the handling of textile waste. Waste from woven polyester–cotton textiles is shredded and then mechanically processed. Subsequently, chemical recycling is applied to the shredded combination.

### 3.3. Thermal Recycling

Textile waste can be converted into activated carbon nanofibers (ACNF) using the thermal decomposition process, which is an effective, financially feasible, and environmentally friendly process [51]. The two crucial processes in this procedure are the acid pretreatment and the following heat breakdown. Textile waste is mechanically shredded into smaller pieces (about 1–2 cm) and submerged in a nitric acid (HNO_3_) solution under reflux conditions during pretreatment [52,53]. This process helps partially dissolve cotton while also assisting in removing colors and contaminants. It is important to highlight that this acidic treatment is a tried-and-true technique widely used in numerous studies intended to qualitatively analyze textile waste [4].

Pyrolysis is an advanced thermal decomposition process utilized for the destructive recycling of textile waste. During this process, the textile material undergoes thermolytic degradation at temperatures ranging from 300 °C to 800 °C, conducted under inert atmospheric conditions to prevent unwanted reactions [54]. Textile waste can be converted into three different types of pyrolysis products: solid, liquid, and gaseous by-products. Char is a solid by-product that is used in electrification and energy generation processes. It is primarily composed of carbon. The high concentration of organic molecules in the liquid by-product, called bio-oil, makes it a possible feedstock for green chemistry procedures. The gaseous by-product comprises combustible gases, some of which have the potential for reuse in recycling initiatives. The char from the pyrolysis is often converted to carbon nanoparticles [55]. Pyrolysis emerges as a recycling technique that effectively converts textile waste into valuable by-products such as char and bio-oil, by dismantling the polymer structure of the feedstock [4]. The kind of feedstock influences the properties of pyrolysis products; however, the pyrolysis properties of textile waste made of synthetic fibers have not received much attention in the literature to date. Mechanistically, this process typically involves chain scission, depolymerization, and volatilization steps. Under high temperature and controlled atmosphere, the polymer backbone experiences random bond cleavage, such as C–C, C–O bonds, by generating radical fragments. These fragments undergo further reactions, such as β-scission, dehydrogenation, cyclisation, and condensation, yielding gases, oils, or waxes. For example, in the pyrolysis of polystyrene, the main mechanism is depolymerization to styrene monomer via backbiting and β scission [56]. The heat-induced breakdown is accelerated by catalysts, heat transfer, and feed composition. The breakdown provides non-volatile char or ash as remnants. Although thermal recycling is complex and produces a broad product distribution, it transforms solid polymer waste into more useful molecules [57].

## 4. Textile Fibers to Micro- and Nano-Fibers and Particles

Textile waste has been transformed into cellulose nanofibers, cellulose nanocrystals, and carbon nanoparticles [58,59,60]. Nanocellulose is generally termed cellulose nanocrystals and cellulose nanofibers. Cellulose nanocrystals (CNCs) and cellulose nanofibers (CNFs) differ in shape, crystallinity, and preparation techniques. CNCs are distinguished by their stiff, rod-like structures, which are produced by chemically acid-hydrolyzing cellulose. In contrast, CNFs are distinguished by their flexible, fibrillar structures, which are produced by enzymatic, mechanical, or alkaline hydrolysis. To clarify the variations in their preparation processes with regard to textile waste, this section distinguishes between these two forms of nanocellulose and carbon nanoparticles.

### 4.1. Cellulose Nanofibers

Textile waste, especially cotton-based garments, can extract cellulose nanofibrils (CNFs) [61]. Mechanical disintegration techniques are commonly used to manufacture cellulose nanofibers by reducing cellulose fibers into nanofibers. This extraction process is often used in combination with chemical or enzymatic pretreatment to improve the cellulose nanofiber yield and size.

Textile waste is first sorted with the focus of separating the cotton-rich waste from the textile material. This separates cotton and non-cellulosic-rich samples, such as synthetic fibers, dyes, and other impurities [62]. Alkaline hydrolysis methods are a bleaching treatment of textile waste samples to remove lignin, hemicellulose, or other textile impurities. Most textile waste may not necessarily contain lignin because it is mainly cotton products, but this is dependent on the source of the textile waste. The product from this process is mainly cellulose, which is needed for conversion into cellulose nanofibers.

**Figure 4:**
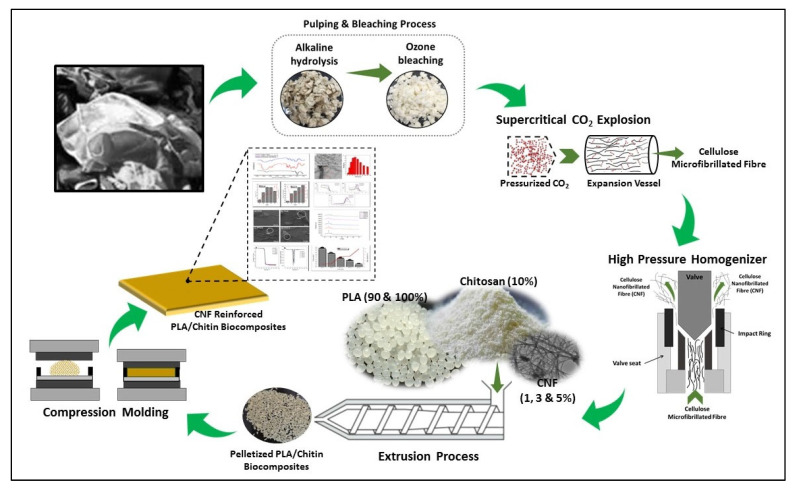
Schematic diagram showing the separation of cellulose nanofiber preparation from polyester–cotton [63].

After pretreatment, the cellulose is broken down into nanofibers using mechanical techniques, including high-pressure homogenization or grinding. Usually, this process produces long, flexible fibrils that range in diameter from 3 to 50 nm [4,62]. Rizal et al. [63] recovered cellulose using an alkaline hydrolysis technique from textile waste made up of 100% cotton garments (Figure 4). The cotton textiles were carefully cut into 2–3 cm pieces using a milling saw. They were then mildly alkaline hydrolyzed with sodium hydroxide (NaOH) to produce cotton pulp fiber. The pieces were heated in a 25 wt.% NaOH solution, augmented with 0.2 wt.% anthraquinone (AQ), at a temperature of 160 °C for 4 h, with all percentages calculated based on the weight of the fiber. Ozone was utilized to bleach the cotton pulp fiber in a non-toxic manner—a chlorine-free approach applied with a gas flow rate of 0.5 L/min maintained at 30 °C throughout the experimental process. The resultant cellulose was further processed into nano-fibrillated cellulose fiber via a supercritical carbon dioxide explosion and high-pressure homogenization techniques. The bleached pulp of cellulose underwent supercritical carbon dioxide explosion for 2 h at a pressure of 500 bars and a temperature of 60 °C, resulting in SC-CO_2_ cellulose micro-fibrillated fiber. Following this, high-pressure homogenization was employed at 56 MPa over a cycle duration of 44 h to yield cellulose nano-fibrillated fiber (CNF). Numerous researchers have conducted studies on the extraction of CNF from textile waste, as summarized in Table 1.

### 4.2. Cellulose Nanocrystal

**Figure 5:**
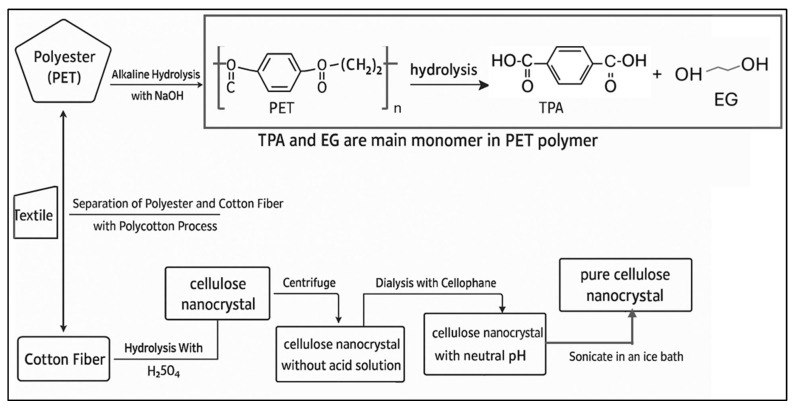
Schematic diagram showing the separation of Nanocellulose preparation from polyester–cotton [4].

**Figure 6:**
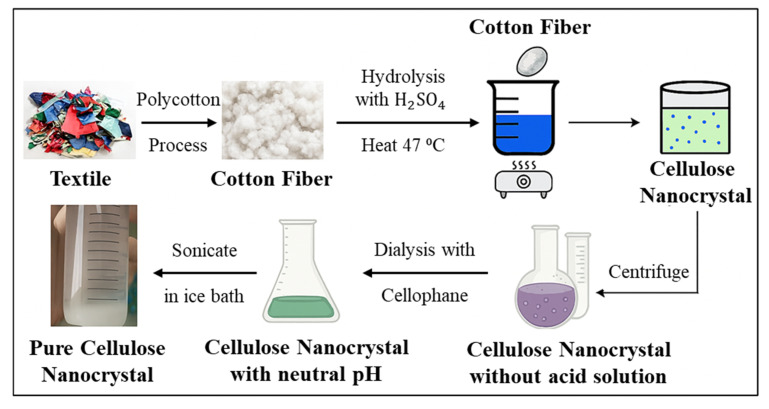
Preparation of nanocellulose from polyester–cotton involves two steps: hydrolysis and subsequent centrifugation and sonication [4].

Cellulose nanocrystals (CNC) are often extracted using acid hydrolysis as opposed to mechanical disintegration techniques used to extract cellulose nanofibers. The production process of CNC is illustrated in Figure 5 and Figure 6. Figure 5 highlights the difference between the two production processes, while Figure 6 shows the schematic diagram for CNC production from textile waste. CNCs, which constitute the initial material, were successfully generated before and after solvent extraction from both raw and pretreated textile waste, utilizing a two-step acid hydrolysis approach. The process comprises concentrated sulfuric acid hydrolysis, followed by the filtration of purified cellulose (PC) employing a 0.5 M sodium hydroxide solution [67]. The degree of polymerization (DP) of PC increased, and the yield decreased after pretreatment, while the side reaction involving CNC formation was inhibited.

CNCs were recovered directly from waste fabrics without undergoing purification processes [4]. The presence of non-cellulosic constituents can result in a high content and irregular shape variations in the residual materials following the separation of CNCs, which may affect the performance of CNCs in composite films. The compatibility and uniformity between nanofillers and the matrix can be improved by altering the surface characteristics of CNCs. Surfactants typically give CNCs a hydrophobic coating, making it easier to dissolve in organic solvents and create different dispersion techniques.

Additionally, a simple sulfuric acid procedure has made notable progress in turning textile waste into cellulose nanocrystals (CNCs) [13,68,69]. Interestingly, CNCs derived from textile waste have the potential to function as a reinforcing agent in biodegradable films [69]. The cellulosic structures of these derived CNCs resemble those of traditional crystalline cellulose, which is effectively made from viscose pulp. The films have a unique water vapor barrier feature because of the CNC structure’s unique porous nature [70]. Additionally, when added to soybean protein isolate films, CNCs from both sources exhibit remarkable mechanical reinforcing qualities and good thermal stability [71].

CNCs were successfully obtained from both cotton and cotton-blended textiles utilizing an innovative technique [58]. Over 85 wt.% of cellulose remained intact following hydrolysis for 90 min in the blended fabric. Results from vibrating sample magnetometry confirmed that textile CNCs exhibited distinctive ferromagnetic characteristics. Up to 75 wt.% of CNCs were magnetically isolated from the matrix of the blended textiles without any dispersing agents. The significant improvement index suggests that textile CNCs could facilitate environmental remediation and textile upcycling due to their inherent ferromagnetic properties, multifaceted performance, and robust thermal stability. Poly/cotton fabric-burned nanocellulose hybrids (FSN) were directly derived from wool and cotton fabrics that were combusted in a furnace at 700 °C. The FSN substrate’s many pores and consequent network structure offer sufficient surface area for the efficient adsorption of contaminants and dyes from wastewater. According to estimated kinetic and equilibrium parameters, the absorption of methylene blue by FSN is primarily controlled by second-order kinetics and multilayer adsorption dynamics. The absorption characteristics of textile hybrid substrates concerning various dyes and pollutants have also been investigated. This work presents a unique approach that avoids the requirement for intricate fabric pretreatment and chemical degradation by directly fabricating active nanocellulose-based porous films with many uses from waste fabrics [67].

The remarkable properties of CNCs, such as their mechanical strength, elevated modulus, higher surface area, unique optical characteristics, sustainability, and biodegradability, make them an important class of natural biological polysaccharides with significant potential across various applications. Research has established methods for isolating CNCs from cotton contained within textile waste and industrial by-products, as presented in Table 2. However, the effectiveness of this isolation process concerning various structures of postconsumer blended fabrics, which can more accurately reflect the characteristics of textile waste, needs to be thoroughly evaluated. Postconsumer fabrics often contain a range of undisclosed chemicals and have been subjected to various chemical and mechanical stresses throughout their lifecycle. The influence of these factors, in conjunction with the structural composition of the fabrics, on the properties of isolated CNCs is not fully understood. Moreover, discarded textiles usually consist of complex blends of various fibers, which causes deterioration to differ among various fabric structures. Even while current research concentrates on creating effective techniques for recovering individual fibers from blended textile waste, it is still unclear if these techniques can enable CNC extraction from cotton without negatively impacting or harming the synthetic components. Despite the successful extraction of colored CNCs from dyed textile waste in other studies, the persistent presence of dye in the final CNC product poses a disadvantage, as it adversely affects the overall properties of the CNCs, particularly concerning the colloidal stability of the CNC suspension. The bleaching procedure is therefore necessary to guarantee the quality and purity of CNCs since this has a major impact on their practical application and financial feasibility. Therefore, a thorough analysis of CNC properties is necessary to determine whether postconsumer textile waste may be used as a source for CNC isolation.

Baloyi et al. [20] examined the properties of CNCs derived from used polyester–cotton waste, with a special focus on how different types of fabric structures impact both the extraction process and the resultant properties of these nanocrystals. A thorough decolorization process was used to produce nanocellulose, which involved treating the waste polyester–cotton textile with hydrogen peroxide and sodium dithionite. The postconsumer material was then heated to 50 °C for 75 min under acid (64% H_2_SO_4_). The percentage yield from their research was up to 69.9%. The excess acid was separated from the mixture using deionized water before ultrasonication to obtain a homogeneous mixture. The authors describe a thorough and systematic decolorization pretreatment procedure that uses hydrogen peroxide and sodium dithionite, which is essential for the effective extraction of cellulose nanocrystals (CNCs). Because it improves the nanocrystals’ quality and purity, this process is essential for ensuring the final product is suitable for later processes. A methodical approach to separating high-quality nanocellulose, which is necessary for its intended uses in composite materials, is demonstrated by the subsequent separation of the CNCs using centrifugation and dialysis against deionized water.

**Figure 7:**
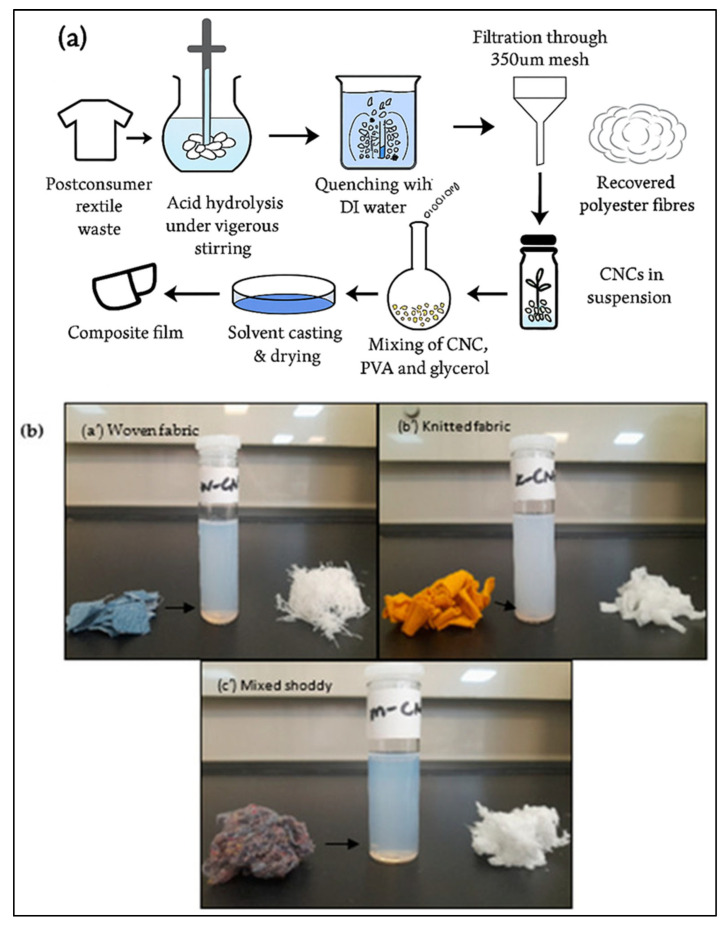
(**a**) Schematic representation of the isolation and separation procedures employed for the extraction of CNCs from postconsumer polyester/cotton waste. (**b**) Photographic documentation of polyester/cotton fabrics prior to processing, alongside the resultant products obtained following acid hydrolysis and filtration [20].

According to the results, the isolated cellulose nanocrystals (CNCs) have a rod-like structure and range in size from 5 to 16 nm in diameter and 78 to 358 nm in length. This morphological feature is important because it affects the CNCs’ mechanical characteristics when they are integrated into polymer matrices. The CNCs’ potential as reinforcing agents in composite materials is further highlighted by their high degree of crystallinity (75–89%) and thermal stability. The practical uses of these nanocrystals in improving the mechanical characteristics and thermal stability of biodegradable films are highlighted by the authors’ study of the incorporation of CNCs with polyvinyl alcohol (PVA) and glycerol to produce composite films. Furthermore, several other authors have successfully extracted CNCs (Table 2), and a schematic diagram illustrating the CNC extraction process is presented in Figure 7.

### 4.3. Carbon Nanoparticles

Carbon nanoparticles extracted from textile waste are a generic way for textile waste recycling. One of the most practiced ways of recycling textiles is burning them, which forms carbon soot. The incineration of textile waste causes environmental pollution; however, a controlled disintegration process using heat can benefit the production of sustainable carbon nanoparticles. Carbon nanoparticles’ exceptional properties, like their high surface area, changing surface chemistry, and electrical conductivity, make them perfect for various uses [86]. The recovery of carbon nanoparticles from textile waste typically entails the utilization of pyrolysis, a thermochemical decomposition process executed at elevated temperatures within an inert atmosphere [87].

The production of carbon nanoparticles from textile waste involves various cutting-edge techniques meant to encourage efficient material transformation and sustainable resource use [88]. The most important of these approaches is pyrolysis, which breaks down cellulose fibers by using heat breakdown in an oxygen-limited environment [88,89]. In this process, long-chain polymers undergo thermal chain scission, including random or specific cleavage of C–C, C–H, and C–O bonds under inert or low-oxygen conditions at elevated temperatures (typically 400–900 °C) and often moderate to high heating rates. The initial step generates free radicals along the backbone, which then undergo β-scission and radical rearrangements that yield smaller volatile fragments (olefins, aromatics, and paraffins), gases (CO, CO_2_, H_2_, and CH_4_), and a residual solid char [90]. Primary fragmentation is followed by secondary reactions of the volatiles, such as cracking, dehydrogenation, cyclisation, aromatization, and also condensation to char [91]. Product distribution depends strongly on temperature, residence time, heating rate, and catalysts. Lower temperatures and faster cooling favor waxes and liquids, while higher temperatures and longer residence times favor gases and char. Pyrolysis enables conversion of polymer waste into fuels and monomer-rich streams, though full selectivity is challenging [91]. This process effectively transforms waste materials into useful carbon nanoparticles by volatilizing chemicals to produce a carbon-rich residue. A number of process variables, such as temperature, heating rate, and retention time, significantly influence the quality and production of final carbon nanoparticles. Enhancing these features allows the final nanoparticles’ properties to be customized for certain applications, boosting their marketability and functional diversity.

An alternative and practical method is hydrothermal carbonization, which uses high pressure and moderate temperature to help turn textile waste into carbon nanoparticles [92,93,94]. This procedure resembles the natural coal creation processes, but it does it much more quickly. Hydrothermal carbonization involves heating wet organic feedstocks in sub- or near-critical water to produce a carbon-rich solid (hydrochar) via a sequence of chemical transformations [95]. Mechanistically, the process begins with the hydrolysis of polymeric or macromolecular material, breaking bonds to yield soluble oligomers and monomers. These soluble fragments then undergo dehydration, decarboxylation, and condensation by forming aromatic or polyaromatic structures that aggregate into the solid hydrochar. Two pathways contribute, including “primary char” from direct solid–solid transformation, and “secondary char” via liquid-phase dissolved intermediates, which repolymerize and precipitate [96]. The resulting hydrochar is enriched in carbon, has lower H/C and O/C ratios, and exhibits enhanced fuel, adsorbent, or soil-amendment properties. Operational parameters, such as temperature, residence time, pH, and water medium, strongly influence the yields and properties [95]. Hydrothermal carbonization is a cleaner production option than traditional incineration or landfill disposal because it captures volatile by-products and condenses organic molecules from textile waste into carbon-rich nanostructures.

The effects of different catalysts and solvent systems are being studied to increase this technique’s efficiency and selectivity, which may result in more control over particle size and surface characteristics. Chemical vapor deposition (CVD) is a high-temperature disintegration of textile waste in a reactor. It is an advanced technology where gaseous reactants deposit a carbon layer onto preselected substrates [97,98]. CVD is often used to prepare high-yield carbon nanoparticles from textile waste, though the procedures are complex. This process can produce high-purity carbon nanoparticles when applied to textile waste, but it usually requires a more complex setup and is more expensive. However, its capacity to produce precise nanoscale features and uniform coatings emphasizes its significance in high-tech applications. The continuous advancement of such production methods raises the potential for recycling textile waste into useful nanomaterials and supports global sustainability goals by lowering environmental consequences and fostering circular economies [98].

A sustainable method of managing textile waste and producing useful carbon materials is to use pyrolysis and hydrothermal carbonization to turn it into carbon nanoparticles. These techniques use controlled thermal degradation of textile waste to produce carbon-rich materials such as carbon nanotubes or nanoballs. The pyrolysis process does not necessitate pretreatments, rendering it a viable method for treating contaminated waste [88,99]. When compared to chemical methods, including biochemical techniques, pyrolysis requires fewer chemical inputs, results in reduced waste generation, and enables faster scalability. In contrast, traditional chemical methods often necessitate more chemicals, produce more waste, and demand considerable time for large-scale operational expansion. Aromatic hydrocarbons represent the predominant liquid products derived from textile waste [88]. The parameters governing pyrolysis operations significantly influence the yield of oil and tar. At a temperature of 500 °C, hydrocarbon compounds and aromatic oxygen were identified. Additionally, alkylphenols were produced at temperatures exceeding 600 °C, while the yield of O_2_ began to diminish at temperatures surpassing 800 °C [89]. At 800 °C, condensable compounds revealed the presence of aromatic compounds devoid of substituent groups, such as naphthalene or benzene. In contrast, aromatic hydrocarbon compounds characterized by three and four rings were produced when the reaction temperature approached 850 °C.

A pyrolysis reactor effectively converts cotton textile waste into carbon nanoballs (CB), which are tiny spherical particles with a diameter ranging from 10 to 20 nanometers, as demonstrated in the research conducted by Yousef et al. [88]. In addition to aiding waste management, this technique produces useful nanoparticles that find use in various industries, such as materials science and electronics. Research by Feng et al. [100] pointed out that the hydrothermal process is a viable substitute for the breakdown of carbon-polymer waste, which is frequently difficult to do with conventional techniques. Based on the temperature ranges, the hydrothermal processing method—which uses water as a reactant—can be divided into five different kinds. Elliott et al. [101] listed these as hydrothermal treatment, hydrothermal carbonization, hydrothermal liquefaction, hot water extraction, and pressurized hot water extraction. Complex organic molecules can be efficiently broken down by the hydrothermal process, which usually runs at a constant temperature near 280 °C.

According to Li et al., the hydrothermal approach has certain disadvantages despite its benefits, such as lengthy reaction periods and the requirement for high-pressure settings [102]. These reasons may complicate the method’s scalability for extensive commercial applications. Furthermore, research conducted by Xu et al. [89] has shown that the hydrothermal carbonization process might be greatly accelerated and enhanced by adding surfactants. This implies that surfactants are crucial for increasing the conversion efficiency of organic matter, which could result in higher yield and higher-quality end products.

**Figure 8:**
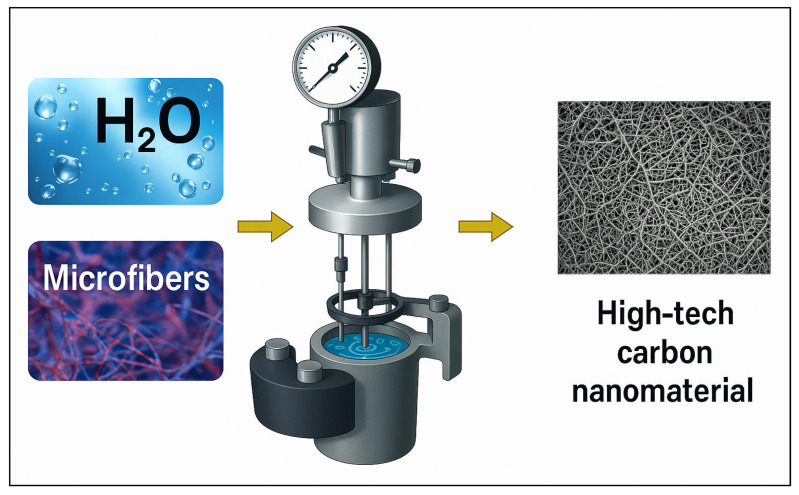
Textile microfibers valorization by catalytic hydrothermal carbonization toward high-tech carbonaceous nanomaterials [93].

The hydrothermal process involves key steps, such as breaking down polyester–cotton fiber waste into smaller particles (Figure 8) and then dispersing this mixture in an aqueous solution with an organic acid catalyst to produce various products [103]. The reaction temperature increased to 140 °C under high pressure, yielding a remarkable 99% recycling yield for aggregates of polyester fiber. Additionally, cotton fiber pieces had a noteworthy 81% recycling yield. In subsequent research, Qi et al. [104] effectively reduced the hydrothermal carbonization temperature to increase hydrophobicity by using FeCl_3_ as a catalyst.

Silvia et al. [93] demonstrated that cotton and polyester can be efficiently transformed into filamentous solid carbon nanostructures by utilizing a Fe-Ni catalyst. Their study demonstrated that a Fe-Ni catalyst can be used to transform cotton and polyester into filamentous solid carbon nanostructures during HTC. The results showed that microfibers from a cotton/polyethylene terephthalate (PET) mixture were transformed into graphitic and amorphous carbon structures, including carbon nanotubes. In every sample, HTC generated graphitic carbon at 200 °C and 22 bar of pressure. This illustrates how altering reaction conditions and catalyst formulation can valorize mixed microfiber trash to produce potentially valuable carbon structures.

## 5. Properties of Textile Waste Nanomaterials

### 5.1. Properties of Nanocellulose from Textile Waste

Textile waste-derived nanocellulose has several interesting physical and chemical properties that make it a promising material for various uses [13]. The remarkable tensile strength, substantial surface area, and high aspect ratio of nanocellulose define its physical characteristics. These qualities come from its crystalline structure, which provides better mechanical qualities than conventional cellulose sources. Because of its large aspect ratio—typically over 100—it works better as a reinforcing agent in composite materials, giving them more strength at a relatively low weight. Furthermore, the enormous surface area of nanocellulose allows for effective chemical changes and interactions with other materials, making it a versatile part of multifunctional systems.

Nanocellulose has many hydroxyl groups, which offer a wide range of chemical modification possibilities [105]. These hydroxyl groups easily participate in processes like graft copolymerization, etherification, and esterification, allowing surface characteristics to be tailored to particular application requirements. For example, by altering its surface, nanocellulose can become more hydrophobic or have better polymer bonding properties.

Nanocellulose’s inherent biodegradability and chemical stability make it a viable substitute for synthetic materials in an environmentally friendly manner, meeting performance and sustainability standards in a variety of industries [3]. Notably, nanocellulose’s ability to create strong hydrogen bonds enhances its usefulness in building resilient networks in aquatic conditions; this characteristic is found in industries such as medicine delivery and water purification. The combination of these physical and chemical properties demonstrates the potential of nanocellulose made from textile waste as a material that can be both sustainable and revolutionary in a variety of industries [105]. Its distinctive properties enable its use in various fields, including creating lightweight, high-strength composites, novel packaging materials, and biomedical devices. This flexibility is particularly helpful in solving contemporary material science issues, such as minimizing environmental impact while enhancing performance features. As research into nanocellulose’s properties progresses, a greater comprehension of its possibilities is expected to encourage innovation and make it easier to incorporate renewable materials into circular economies.

**Figure 9:**
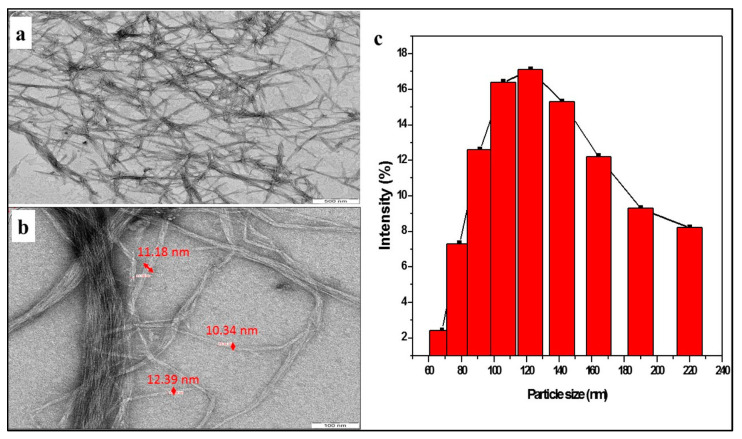
(**a**,**b**) TEM images of cellulose nanofibers from recycled textiles and (**c**) particle size distribution [63].

Figure 9a,b present transmission electron microscopy (TEM) images of cellulose nanofibers, which exhibit diameters in the nanoscale range of 10 nm to 30 nm, as calculated using the diameter measurement functionality of the TEM software. Further validation of these diameter measurements was performed with ImageJ software (https://imagej.en.softonic.com/, accessed on 20 January 2021). Notably, the nanofibers display interconnectivity, as depicted in the figure [63].

The particle size distribution of the separated cellulose nanofibers is shown in Figure 9c. The intensity (%) of each particle size is plotted in accordance with the particle sizes, which are shown on a percentage scale. According to the particle size study, there is a noticeable peak between 100 and 120 nm, ranging from 60 to 220 nm. This outcome supports the successful extraction of nano-fibrillated cellulose fibers from textile waste since it shows a sizable percentage of nanofibers localized in the 100 nm to 120 nm range.

**Figure 10:**
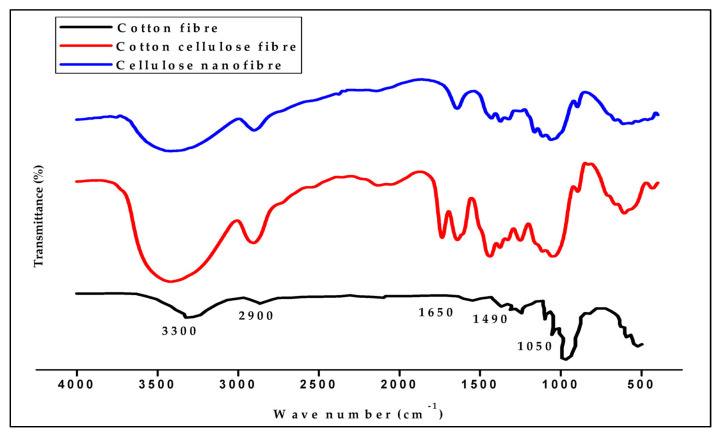
Fourier-transform infrared spectroscopy (FT-IR) analysis of cellulose nanofibers extracted from textile waste fabrics [63].

The FT-IR graph presented the absorption spectra for a typical bond found in cellulose nano-fibrillated fiber. The absorption band between 2500 and 3000 cm^−1^ represents the C–C bond, whereas the strong peak between 3200 and 3500 cm^−1^ represents the hydroxyl (OH) bond. Furthermore, the C–H bond is identified by the spectrum region between 1500 and 2000 cm^−1^, while the C–O stretching vibrations are represented by the peak at 1050 cm^−1^ [63]. The band located between 400 and 800 cm^−1^ is commonly recognized as the amorphous band of nanocellulose. As shown in Figure 10, these detected peaks are typical of the chemical linkages inherent to nanocellulose. The investigation shows that the final product’s chemical structure remains unchanged when chemicals are added during the cellulose nanofibrils (CNF) isolation process. Additionally, there is a good agreement between the FTIR graph and data obtained using other analytical techniques reported in the literature. Notably, the –OH stretch band exhibited a reduction in absorbance as the raw fiber approached complete isolation of CNF. Importantly, new bands are absent following the CNF isolation process, suggesting that no new chemical bonds were formed with the hydrolysis chemicals, which implies that the cellulose production process does not entail digestion or bleaching [63].

### 5.2. Properties of Carbon Nanoparticles from Textile Waste

Textile waste-derived carbon nanoparticles, a comprehensive understanding of their properties and behaviors is paramount for leveraging their potential across a multitude of applications [97]. These nanoparticles’ unique physical, chemical, and mechanical properties set them apart from traditional carbon materials. This is mostly because of their nanoscale size and the intrinsic properties of carbon generated from textiles [106]. Physiologically, carbon nanoparticles have high surface area-to-volume ratios that boost their ability to interact with the environment. This makes them desirable candidates for applications that require better conductivity, adsorption, or catalysis [107]. The reactivity and compatibility of these nanoparticles in composite materials or chemical processes are influenced by the fact that they frequently retain functional groups from the precursor textile waste. Most nanoparticles’ hydrophilic or hydrophobic properties can be altered by reacting oxygen-containing functional groups with materials. This can be used to enhance their dispersion in the matrix material.

Additionally, the synthesis conditions and subsequent processes affect these carbon nanoparticles’ thermal stability and degradability [107]. A thorough comprehension of these properties is essential for maximizing the effectiveness of nanoparticles in certain applications, including electronic devices, filtration systems, and reinforced materials. The way carbon nanoparticles interact at the molecular and atomic level determines how they behave in various settings. Their high strength-to-weight ratios and other mechanical characteristics make them especially important for applications requiring impact resistance or load-bearing capabilities. At the same time, their electrical properties—which are characterized by unique conductive qualities—offer the possibility of developing sensors and electronics. However, the complexities of these behaviors are contingent upon factors such as particle size distribution, aggregation tendencies, and inter-particle forces, all of which are affected by the conditions of synthesis and the initial textile waste material. In conclusion, the intricate properties and behaviors of textile waste-derived carbon nanoparticles position them as versatile materials capable of driving innovations across various fields.

**Figure 11:**
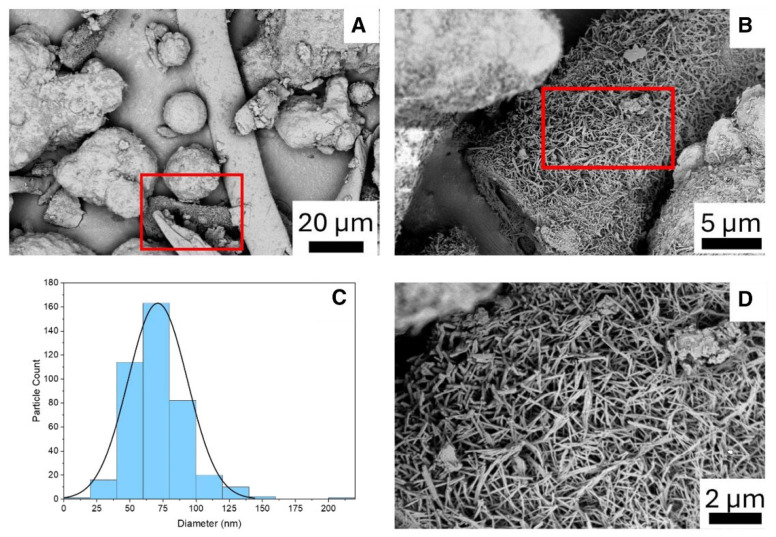
Carbon nanotube structures produced from PET/cotton following catalytic HTC reaction at 200 °C and 12 h of residence time with different magnifications (**A**–**C**). (**A**) delimited possible CNT clusters, (**B**) delimited area from (**A**), and (**D**) delimited area from (**B**) with the diameter distribution at (**C**) (Data are represented as mean ± 2.35) [93].

Figure 11 illustrates potential synthesis methods for carbon nanotubes resulting from the catalytic HTC reaction of PET/cotton at 200 °C for 12 h.

## 6. Environmental Applications of Nanomaterials Derived from Textile Waste

Traditional textile manufacturing methods often raise serious environmental issues, especially regarding heavy metal contamination and coloring methods. To address these problems, scientists have concentrated on applying nanocellulose in the textile industry, looking at how it may be utilized in filtration procedures to remove heavy metals and colors from wastewater. Given that the textile industry is a significant cause of water pollution due to the discharge of dye-laden effluents, the extraction of dyes from textile wastewater has become a crucial issue in the modern environment. Conventional color removal techniques, such as chemical oxidation, adsorption, coagulation, and precipitation, are often ineffective and costly and frequently produce large amounts of sludge [108,109]. By successfully absorbing and removing dyes through both physical and chemical interactions, nanocellulose-based filtration techniques, on the other hand, offer a promising substitute and the possibility of more effective and environmentally friendly dye removal procedures [108].

Amiralian et al. [110] demonstrated a viable method for removing dyes from wastewater. Using cellulose nanofibers as templates, the study concentrated on creating magnetic nanoparticles, which produced uniformly sized particles with diameters less than 20 nm. Then, using in situ hydrolysis of metal precursors at room temperature, these magnetic nanoparticles are affixed to the nanofiber surfaces. The study examines how different nanofiber concentrations affect the final membranes’ shape, crystallite size, and thermal and magnetic characteristics. The efficiency of the produced magnetic membranes is confirmed by their remarkable magnetic capabilities and superparamagnetic characteristics, which are enhanced by their high density of magnetic nanoparticles. Interestingly, within 300 min at ambient temperature, these magnetic membranes significantly degrade rhodamine B, a hydrophilic organic dye often used in many different sectors, by 94.9%. The effective synthesis of magnetic nanoparticles using cellulose nanofibers is demonstrated in this work, which offers important new information on the processes behind nanoparticle development and creation.

Anionic dyes hurt the environment and pose health risks, making their effective removal from industrial effluents a major challenge in wastewater treatment. Bassyouni et al. [111] removed anionic dyes from textile industry effluent using nanocellulose, marking a significant breakthrough in environmental remediation. To make nanocellulose/chitosan nanocomposites, nanocellulose derived from palm leaves was used. Utilizing batch experiments, the study assessed the effectiveness of chitosan, nanocellulose, and the novel synthetic nanocellulose/chitosan microbeads (CCMB) to eliminate direct anionic blue 78 dye. Adsorbent concentration, mixing duration, pH, and starting dye concentration were among the many factors considered. The synthesized CCMB was notable for having a positive net surface charge and a surface area of 10.4 m^2^ g^−1^. Maximum efficiencies of 91.5% and 88.4% with CCMB ratios of 0.25:1 were obtained in the adsorption tests, demonstrating a clear relationship between adsorbent concentration and dye removal efficiency. The researchers found that the best clearance efficiency was obtained with a nanocellulose/chitosan ratio of 0.5:1. When combined, chitosan, nanocellulose, and CCMB showed promise as effective adsorbents for wastewater treatment, especially in the removal of dyes.

On the other hand, there are serious environmental concerns about heavy metals in textile effluent. Often found in textile finishes and dyes, heavy metals can hurt ecosystems and human health [112]. The selectivity and reusability of conventional heavy metal removal techniques, such as membrane filtering, electrochemical treatment, and precipitation, are limited [113,114]. Filtration methods based on nanocellulose show great promise for overcoming current constraints by using the large surface area and adsorption ability of nanocellulose to remove heavy metal ions from textile effluents in a targeted manner.

Recent developments in the study of nanocellulose have shown promise as a novel adsorbent in the water treatment industry. Notably, Li et al. researched the removal of heavy metals using TEMPO-oxidized cellulose nanofiber (TOCNF) [115]. TOCNFs have proven to be highly effective at removing Cu (II) and Zn (II) from aqueous environments because of their exceptional capacities and quick adsorption kinetics. Their selective affinity demonstrates these materials’ specificity and efficacy for copper adsorption. Their potential importance in sustainable water reclamation efforts is further supported by the clarification of intricate adsorption mechanisms, including ion exchange, coordination, and accumulation.

Aerogels based on cellulose nanofibril (CNF) have become well-known as a sustainable and effective method of removing heavy metals from wastewater, as these advancements have occurred. Tang et al. [116] produced cellulose nanofibril (CNF) aerogel sorbents, a sustainable nanomaterial, to eliminate a variety of pollutants from wastewater. CNFs were cross-linked with polyethyleneimine (PEI) to create the aerogels, and polydopamine was applied to their surface using a coating technique inspired by mussels. In order to create a strong porous structure, the synthetic process was streamlined to use the fewest possible raw ingredients. The aerogels had a high porosity of 98.5%, a low density of 25.0 mg/cm^3^, and the ability to restore their shape in both water and air. Cu (II) and methyl orange (MO), two representative pollutants, were the subjects of adsorption investigations. The intra-particle diffusion model could explain the adsorption mechanism, and the kinetic data followed the pseudo-second order kinetic model. The maximal adsorption capacities for Cu (II) and MO were determined by fitting the Langmuir model to be 103.5 mg/g and 265.9 mg/g, respectively. It was determined that the aerogels can sustain a high adsorption capacity across a broad pH range by evaluating the impact of pH on the adsorption performance. These aerogels’ notable adsorption capacities demonstrate their potential as workable solutions for environmentally friendly wastewater treatment. These results highlight the adaptability of nanocellulose in tackling intricate problems in the textile sector, especially the elimination of colors and heavy metal ions.

Most of the materials and methods used in environmental control and cleanup are outdated, posing several health and environmental risks to living things. For example, non-biodegradable materials are used in combination with transition metal-based adsorbents and catalysts to remove a variety of harmful pollutants. However, these materials pose serious threats to the local environment since they can emit hazardous metal ions and non-biodegradable by-products, which can lead to secondary contamination. As a result, researchers are concentrating more on creating and promoting “green” and “biodegradable” materials for environmental restoration, which could reduce primary and secondary pollution.

There are several benefits and drawbacks to the current technologies for the removal of various pollutants, such as heavy metal ions, dyes, polymers, detergents, and pesticides, in terms of both cost and efficacy. As a result, continuous progress in this field is essential. Because of its renewable, biodegradable, and environmentally benign qualities, advanced nanocellulose (NC)-based water treatment and purification technologies are becoming more popular than alternative techniques. Numerous NC-based nanomaterials have been thoroughly investigated for possible uses in environmental cleanup within the last ten years. Since these materials require less energy and have a low propensity to produce hazardous by-products, Ul-Islam et al. reviewed numerous studies demonstrating their superior pollutant removal efficacy, supporting their use as the best option for environmental protection and rehabilitation [117].

The surface properties of nanocellulose materials greatly aid the removal of aquatic contaminants, whether natural, modified, or customized. Innovative materials based on nanocellulose (NC) rely on their intrinsic properties, such as surface area, active sites, density of functional groups, and other relevant features, to be effective. NC can be used in reactive material templates, membranes, or solid fiber networks, among other applications in the field of pollution remediation. Furthermore, the methods used to purify water greatly influence how much pollution is removed, especially when cellulosic hybrid materials are used. The parts that follow go over NC-based materials and the methods used for water treatment.

There is a lot of scholarly interest in studying nanocellulose as a filter for various viruses [118]. Many viruses, including COVID-19, are categorized as airborne pathogens that can spread through respiratory droplets from infected people’s saliva or nasal secretions. Therefore, an effective, long-lasting, and reasonably priced air filtration system that reduces the dangers of airborne virus infection is desperately needed. This kind of air filtering solution has been the subject of numerous recent research articles. Filter thickness, pore size, number of layers, virus dimensions, filter surface charge, ionic strength, and surface chemistry are some factors that affect how effective the air filtration process is. Size-exclusion filtration techniques generally offer several benefits, such as adaptability and operational ease, since they remove viruses predictably based on physical traits, make it easier to filter viral markers for simple filtration process validation, and do away with the need for dangerous or mutagenic chemicals in viral inactivation processes.

Gustafsson et al. [119] evaluated a membrane filter constructed from nanocellulose, arranged in a mille-feuille configuration with varying thicknesses, utilizing a simulated wastewater matrix to assess its efficacy in virus removal for drinking water purification applications. The filtration processes involved samples of simulated wastewater, which contained total suspended solid content represented by 30 nm latex particles used as surrogate waste material and 28 nm ΦX174 bacteriophages serving as the viral contaminant. The authors investigated the performance of these membrane filters under pressures of 1 and 3 bar, with thicknesses ranging from 9 to 29 µm. The results indicate that a membrane filter composed of 100% nanocellulose can effectively eliminate even the smallest viral particles, achieving removal efficiencies between 99.9980 and 99.9995%.

Manukyan et al. [120] assessed the viability of employing mille-feuille filter paper based on nanocellulose for upstream uses in serum-free growth media filtration, namely Dulbecco’s modified Eagle’s medium (DMEM) and Luria-Bertani medium (LBM). As a model small-sized virus, the filter’s ability to remove the ΦX174 bacteriophage (28 nm) was evaluated for filters with different thicknesses (11 and 33 µm) and operating pressures (1 and 3 bar). With LRV ≥ 5, the filters showed generally good model small-sized virus elimination characteristics, particularly for 33 µm filters. Despite having a usually lower flow, the 33 µm filters were more resilient and had superior viral elimination and throughput characteristics compared to the 11 µm filters. The efficacy of the 33 and 11 µm nanocellulose-based filter sheets for upscaled bioprocessing was further confirmed by increasing the loading capacity by ten times. Consequently, the findings of this research suggest that the nanocellulose membrane filter presents a viable alternative for the filtration of substantial volumes of cell culture media in upstream processes.

Asper et al. [121] confirmed that size-exclusion filter paper made entirely of naturally generated cellulose could effectively remove the xenotropic murine leukemia virus (xMuLV). Cellulose nanofibers from *Cladophora* sp. algae were used to make filter paper. Atomic force microscopy, scanning electron microscopy, helium pycnometry, and model tracer (50 nm gold nanoparticles and 100 nm latex beads) retention tests were used to describe the filter paper. The tissue infectivity test’s sensitivity and the virus titre in the feed solution determined that LRV ≥ 5.25 log10 TCID50 was noted after the filtration of xMuLV-spiked solutions. According to the validation study’s findings, endogenous rodent retrovirus and retrovirus-like particles can be eliminated throughout the recombinant protein manufacturing process using nanocellulose filter paper.

Metreveli et al. [122] worked on designing virus removal membrane filters, presenting a significant challenge, particularly in tailoring the upper pore size cut-off. It is crucial to retain viruses, which typically have particle sizes ranging from 12 to 300 nm, while simultaneously allowing the unobstructed passage of proteins, which generally range from 4 to 12 nm in size. High porosity is therefore required for nanocellulose-based filters in order to address the problem of poor permeance. Researchers created a 70 μm-diameter nanocellulose membrane filter with a 35% overall porosity for their study. With a particle size of 80–120 nm, the Swine Influenza A Virus (SIV) was successfully eliminated by this filter [122]. The effectiveness of the design was further supported by the authors’ observation that latex beads and SIV particles appeared as layered formations on the porous membrane filter’s surface. It was also discovered that proteins may flow through this membrane filter without any problems. Consequently, this study’s pore size distribution shows potential for virus filtering applications, especially for bigger viruses that are larger than or equivalent to 50 nm.

Besides this, Mautner et al. [123] produced BNC membrane filters with higher porosity to maximize permeability and reject contaminants at the nanoscale. Before the membrane filter, the BNC was treated with organic solvents such as ethers, ketones, and alcohols. The porosity of the produced treated BNC membrane filter was 67%, which was higher than the 33% porosity of the untreated BNC membrane filter. Due to the decreased membrane density, this treated membrane filter exhibits a 40-fold increase in permeability. Attributable to its decreased membrane density, this treated membrane filter also exhibits a 40-fold increase in permeability. The produced membrane filter notably keeps pore diameters between 15 and 20 nm, similar to the untreated BNC membrane filter, even with the increase in porosity. As a result, under high flow circumstances, the improved membrane filter supports the efficient removal of viruses via a size-exclusion mechanism.

Nanocellulose’s strength is essential for creating membrane filters that effectively eliminate viruses using a size exclusion mechanism. In a study by Quellmalz and Mihranyan et al. [124] found that membrane filters based on citric acid cross-linked nanocellulose perform better mechanically than untreated nanocellulose. Its potential industrial applications are limited by observations that show the untreated nanocellulose membrane filter is susceptible to cracking at pressure gradients greater than 15 kPa. Higher pressure gradients can be applied during filtration thanks to the cross-linked nanocellulose membrane filter’s increased strength without endangering the filter’s structural integrity. Thus, it can be said that the cross-linking of nanocellulose with citric acid offers substantial benefits in several industrial applications targeted at virus elimination.

The filtering performance against viruses has improved because of earlier studies on the surface modification of nanocellulose. As was previously mentioned, the addition of quaternary compounds greatly improves the electrostatic contact between viruses and nanocellulose. For example, viruses such as coronaviruses possess negatively charged surfaces, which enable them to interact effectively with either the cationic or anionic charges derived from nanocellulose-quaternary compound combinations.

The entrapment of the virus within a nanocellulose matrix occurs due to the electrostatic attraction between the negatively charged virus particles and the positively charged nanocellulose membrane. Several studies have shown that using cationic chemicals to filter negatively charged viruses can produce positive results. Mi et al. [125] reported a filtration system that uses a modified CNC with a positively charged guanidine group to entirely remove the Sindbis and porcine parvoviruses from water by adsorbing them. Notably, this filtration system surpasses the virus removal standards set forth by the Environmental Protection Agency (EPA) for potable water. Furthermore, the system leverages the electrostatic interactions between the viruses and the guanidine group to enhance its efficacy. Additionally, Meingast and Heldt [126] stated that “the complete removal of viruses from water is attributed to the protonated guanidine groups present on the cationic cellulose nanocrystals (CNC), which facilitate the formation of both ionic and hydrogen bonds with the proteins and lipids found on the virus”.

Other than that, Rosilo et al. [127] investigated the significant affinity binding between cationic cellulose nanocrystals (CNC), specifically the CNC-g-P(QDMAEMA) mixture, and the cowpea chlorotic mottle virus (CCMV) as well as norovirus-like particles in aqueous dispersions. Notably, this cationic CNC mixture was synthesized through surface-initiated atom-transfer radical polymerization of poly (N,N-dimethylamino ethyl methacrylate), followed by a quaternization process addressing the polymer’s pendant amino groups. Furthermore, the anionic CCMVs were successfully removed using functionalized lignin containing a quaternary amine. The observations from the study revealed that CCMVs formed agglomerated complexes with cationic lignin. This finding indicates the potential application of this material in the development of membrane filters aimed at the removal of CCMVs.

Besides that, Sun et al. [128] found that the EV71 and Sindbis viruses were effectively filtered by covalently altering cellulose nanofibers (CNF), particularly by functionalizing them with polyglutamic acid (PGS) and mesoporous silica nanoparticles (MSNs). The interaction between the negatively charged MSNs on the modified CNF and the positively charged amino acids histidine (His10) and lysine (Lys14) is responsible for this effectiveness.

Numerous studies have been conducted on the use of nanocellulose as a filtration medium for the removal of microorganisms. The average diameter of waterborne bacteria is greater than 0.2 μm [31], which, via the size-exclusion process, makes it easier for membrane filters based on nanocellulose to effectively capture a variety of bacterial species. Additionally, it is possible to improve the characteristics of nanocellulose by surface functionalization, which would increase the effectiveness of bacterial elimination.

Wang et al. [129] proved that a multilayered nanofibrous microfiltration system with high flux, low pressure drops, and remarkable retention capabilities against bacteria—specifically, *Escherichia coli* and *Brevundimonas diminuta*—was feasible. An electrospun polyacrylonitrile (PAN) nanofibrous scaffold supported by a poly (ethylene terephthalate) (PET) nonwoven substrate was impregnated with ultrafine cellulose nanofibers (CNF) to achieve this breakthrough. The TEMPO oxidation technique was used to functionalize the CNF with carboxylate and aldehyde groups prior to impregnation. The CNF-based microfiltration membrane demonstrated full retention capability against bacteria, according to observations.

Otoni et al. [110] developed a cationic cellulose nanofibril (CNF) compound utilizing Girard’s reagent T (GRT). The researchers shaped this compound into foam through various protocols, including cryo-templating, aimed at eliminating the prevalent human pathogen *Escherichia coli*. The porosity of this foam, which is directly correlated with its surface area and pore size, plays a critical role in the effective removal of *Escherichia coli*. The cryogel foams produced via this methodology exhibited porosities of approximately 98% and demonstrated an estimated 85% higher anti-*Escherichia coli* activity when compared to control samples composed of unmodified CNF. The cationic CNF generated using GRT illustrated considerable potential for both air and liquid filtration applications, showing remarkable absorbency facilitated by functional coating. Access to safe drinking water has emerged as one of the paramount challenges globally, impacting both high- and low-income countries, particularly as natural resources become increasingly scarce.

Gouda et al. [130] modified an electrospun cellulose nanofiber (CNF) membrane integrated with silver nanoparticles (AgNPs), which was developed as an innovative water disinfection system aimed at enhancing water purification processes. The AgNP concentration, surface shape, physical characteristics, and antibacterial activity of the resulting membrane filter were all thoroughly investigated in the study. AgNPs, which are classified as biocidal nanoparticles, have significant antibacterial qualities that are mostly explained by their size quantization effect, which affects metal reactivity at the nanoscale. The resulting membrane filter demonstrated exceptional efficacy in eliminating bacteria, including *Escherichia coli*, *Salmonella typhi*, and *Staphylococcus aureus*, achieving a filtration efficiency exceeding 91% in contaminated water.

Ottenhall et al. [131] developed a cellulose nanofiber (CNF)-based membrane filter, modified with polyelectrolyte multilayers, which resulted in the production of multilayer cationic polyvinyl amine (PVAm) and anionic polyacrylic acid (PAA). The authors have successfully modified the CNF with the cationic polyelectrolyte PVAm, as well as the anionic polyelectrolyte PAA, in configurations involving either single layers or multilayers (comprising three or five layers) through a water-based process conducted at room temperature. The functionalized CNF-based membrane filters, which include several layers, can eliminate more than 99.9% of *Escherichia coli* from aqueous solutions, according to filtration analysis. In contrast to traditional processing methods that use plain nanocellulose filters, the three-layer membrane filter showed an impressive performance in removing cultivatable bacteria from natural water samples, with an efficiency of over 97%.

## 7. Challenges and Future Research

Textile waste, especially from postconsumer garments, imposes a significant burden on landfills and embodies a substantial waste of both natural and synthetic resources. This offers a chance to implement efficient waste extraction techniques and create a circular economy [20,67]. The substantial amount of textile waste and the difficulties posed by modern confinement materials, different colors, and enormous product dimensions make resource recovery from textiles challenging [132]. Recent developments have made it possible to use a two-step alkaline-oxidative technique to extract cellulose nanocrystals (CNCs) from postconsumer polyester–cotton mixed fabrics [4]. Although various textile recycling methods are available, most do not preserve fiber length or facilitate the reuse of fibers in composite materials. The input materials, fiber type, size of the resulting flakes, and general waste disposal strategies all significantly impact the particular recycling techniques used [38].

One of the world’s biggest problems today is the incineration and landfilling of textile waste. Globally, more than 100 billion garments are produced each year. In North America, an estimated 10 million tons of textile waste are disposed of in landfills [67]. This practice is unsustainable, as continuously dumping waste into confined land will inevitably trigger a waste disposal crisis. Furthermore, it causes more serious problems, including land scarcity and leachate-induced soil pollution, which is frequently poisonous and cancer-causing [133]. The waste produced by the textile industry is an indicator of resource misallocation and environmental deterioration. Natural fibers, particularly cotton, which contain over 90% cellulose, comprise about one-third of textiles. Polyester, on the other hand, is a synthetic fiber made from petroleum-based chemicals [134]. Cotton textile waste exhibits a biodegradability of over 80%, thereby facilitating a closed carbon cycle and minimizing environmental pollution [135]. On the other hand, polyester fabrics provide considerable environmental danger; yet, they may be recycled to produce chemicals and fuels, albeit with energy-intensive techniques and high temperatures [48]. Proactive steps should be taken to stop textile waste from entering landfills and incinerators in the first place in order to address the environmental issues related to textile waste, such as accumulation, the leaching of hazardous chemicals, and declining air quality. Initiatives have been undertaken to recycle textile waste into new fibers, such as worsted textiles, and to repurpose it for trading and manufacturing cleaning rags, thereby reducing disposal into landfills. Currently, only 20% of postconsumer clothing is recycled; the remainder is either downcycled or remains untreated [136].

Converting large volumes of textile waste into micro- and nano-sized particles appropriate for various uses presents several technical difficulties. The different petrochemical and natural fiber types that make up textiles have unique structural properties and a great chemical variety. When creating nanoparticles from bigger macro-sized materials, this heterogeneity poses significant challenges. For instance, electrospinning requires an appropriate solubility or melting point to turn micro-sized polymer fibers into homogeneous polymer nanofibers efficiently. It is crucial to create reliable methods to convert various textile waste into materials that are micro- and nano-sized to overcome these technical obstacles [67].

Textiles’ initial economic worth has increased significantly due to treatment, supporting the established process’s economic feasibility. According to the comparison analysis, the entire value of textiles is lost when recycling methods are not implemented, which has a negative effect (−R/7.6). On the other hand, research on the synthesis of viscose nanofibers from textile waste showed a positive economic outcome (R/1.81). This methodology’s important facets merit more investigation in subsequent research. Interestingly, the T0 treatment increased value without adding to expenses, especially by using composted waste, indicating a variety of setups for different bio-recycling techniques. Furthermore, it is essential to examine the expenses associated with the transportation and shredding of textile waste before its conversion to viscose nanofibers, particularly in relation to the current recycling facilities available within Brazil [14]. Since the analysis focused on a different kind of textile waste, the shredding costs significantly impact the findings. Future studies should factor in these costs to allow for a comparison of results.

The valuations of cotton and viscose nanofiber production were predicated on values expressed in US dollars. To achieve comparability with previously published studies, the analysis of nanofibers produced from textile fibers also incorporated values in US dollars. The assessment of non-treated textile waste processing, priced at US$0.50 per kg, was derived from treatment practices in developed nations [137]. Such analyses must be conducted in less economically stringent environments to ascertain the influence these conditions may exert on the results obtained. Furthermore, subsequent research should evaluate the impact of soil valorization on the sustainability of treatment methodologies. Given the need to reduce the environmental effects of processing textile waste in relation to the types of textile fibers substituted, a more thorough analysis of the range of these variables is necessary. The possible directions for further research are explored with an emphasis on the difficulties related to sustainable cellulose nanofibers in the textile waste industry. First, there is still difficulty with mixed materials, such as blends of polyester and cotton, polyester and viscose, and others. This persists despite progress made in alkaline treatments of cotton and the biodegradation of both viscose and polyester, highlighting the necessity for further foundational knowledge regarding synergistic eco-friendly treatments. Moreover, nonwoven materials generate voltages that are insufficient for directly powering a pump, which leads to engineering design complications, as empirical evidence of process efficiency remains unproven. The research domain concerning the slow degradation of biodegradable polymers, including natural polymers, continues to present opportunities for exploration.

Textile waste has become one of the biggest environmental problems in recent decades. Discarded clothing, production waste from the fabric and garment manufacturing industry, and used goods that are no longer needed or in acceptable shape are the main sources of this trash. Around 920 billion new garments were sold worldwide in 2018 alone, and estimates suggest that the number could rise to 1 trillion by 2030. Initiatives encouraging domestic recycling through institutional programs and donations are gaining steam, despite the concerning figures that one-third of purchased clothing eventually ends up in landfills. A comprehensive strategy that incorporates recycling techniques from garment to garment, fabric to fabric, and fiber to fiber is required to address circularity in the textile industry successfully. Investigating sustainable recycling methods for cellulose-based textiles and the related sustainability concerns has been the focus of recent studies [9].

Most textile recycling studies have focused on cotton and viscose-based materials; on the other hand, there are very few studies on polyester-based recyclables, even though polyester is a significant component of many textiles. Because it is difficult to source virgin sugar from polyester in a way that is both non-toxic and environmentally acceptable, there has not been much discussion about recycling polyester. Furthermore, the extraction of alternative biobased recyclable materials, including natural cellulose, has frequently received scholarly interest [4]. Recycling processes that involve biodegradation necessitate a comprehensive understanding of the inherent low biodegradability of synthetic polyesters within natural ecosystems. This evaluation elucidates the effects of mixed waste on cross-linked polymers following processes of melting and molding, while also proposing strategies for the reduction of textile waste. The shift to a sustainable circular textile economy may be facilitated by the textile industry’s advancement of alternatives, including biobased materials and plastic substitutes, especially by highlighting both biobased and biodegradable possibilities. However, the textile business must be rigorous in tracking down and being transparent about the resources used across the whole value chain of purchased textiles. Furthermore, it is necessary to provide an update on the most recent environmental evaluations and regulatory actions related to the textile industry.

## 8. Conclusions

Recycling techniques and sustainable materials science have advanced significantly by converting textile waste into nanocellulose and nanoparticles. Using novel techniques for breaking down textile waste, scientists clarify the possibility of turning waste fiber into useful products. In addition to addressing the pressing issue of textile waste management, this innovative approach reveals many possibilities in materials engineering, electronics, and pharmaceuticals. The viability of creating a circular economy based on textile materials is becoming increasingly likely as these technologies progress. However, there are many obstacles to the adoption of these recycling procedures. While cotton fibers offer biodegradability, polyester remains a major concern, requiring energy-intensive recycling. Recent advances, such as alkaline-oxidative methods for extracting cellulose nanocrystals from mixed fabrics, show promise for circular economy initiatives. However, technical and economic barriers persist, including fiber heterogeneity, shredding costs, and process efficiency. To mitigate pollution and resource loss, proactive strategies for fiber recovery, sustainable recycling, and value-added conversion into micro- and nanomaterials are essential, alongside further research into eco-friendly treatments and scalable technologies. Economic viability, the requirement for effective and scalable extraction methods, and the creation of regulatory frameworks that encourage such advances are also key challenges that need addressing. To overcome these obstacles and create sustainable practices that ease the shift from linear to circular economies, industries, governments, and researchers must work together. A more sustainable future might be promoted, and environmental effects could be lessened if nanocellulose and nanoparticles made from textile waste are successfully realized.

## Figures and Tables

**Table 1:** Cellulose nanofibers from textile waste.

Type of Textile Waste	Method	Shape/Size	Yield (%)	Reference
100% postconsumer cotton fabric	Alkaline hydrolysis+ supercritical CO_2_ + high pressure homogenization	Typical rod-shaped structure 100–120 nm	Not specified	[63]
Waste cloth made of cotton material	H_2_SO_4_/HCl hydrolysis	- Length range: 25.38 nm. - Crystallinity percentage: 56.17%.	77	[64]
Waste polyester–cotton fabric	Cellulose extraction via alkali, decolorizing, and hydrochloric acid treatment followed by H_2_SO_4_ hydrolysis.	Not specified	56	[4]
White bleached cotton fabric	TEMPO-facilitated oxidation for producing CNF	- Length: 175.2 nm - Width: 11.4 nm - Crystallinity: 71.6%	73	[65]
Indigo-dyed denim fabric.	TEMPO-facilitated oxidation for producing CNF	- Length: 162.3 nm - Width: 9.90 nm - Crystallinity: 66%	79	[65]
Waste cotton fabric	Combined two-step process, involving electron beam irradiation.	Low-charged cellulose nanorods Rod-like shape Length: 50–300 nm Aspect ratio 18.4	90	[66]

**Table 2:** Cellulose nanocrystals from textile waste.

Type of Textile Waste	Method	Size	Yield (%)	Reference
Waste cotton fabric	Alkaline bleaching and acidic hydrolysis	Length ranges from 28 to 470 nm, with a diameter between 3 and 35 nm.	47	[72]
Denim fabric	Acid hydrolysis, along with mechanical grinding	Length: 214.6–234.6; Diameter: 23.7	90–98	[73]
White bleached cotton fabric	Chemical processes, including acid hydrolysis and TEMPO oxidation, as well as mechanical methods.	- Length: 127.7 nm - Width: 11.9 nm - Crystallinity: 86.4%	40	[65]
Denim fabric dyed with indigo	Chemical processes, including acid hydrolysis and TEMPO oxidation, as well as mechanical methods.	- Length: 151.7 nm - Width: 12.5 nm - Crystallinity: 85.6%	38	[65]
Cotton by-products	Chemical methods (oxidation bleaching, acid hydrolysis) and mechanical processes.	50 nm	-	[74]
Cotton waste from industrial processes	Acid hydrolysis (chemical process)	Length: 180 ± 60 nm Diameter: 10 ± 1 nm	45	[71]
Viscose fiber textile waste	Chemical oxidation via APS	34–49 nm	38–40	[75]
Denim Waste	Chemical (alkaline treatment, APS oxidation)	Length: 76.14 ± 8.56 nm, Diameter: 18.10 ± 3.54 nm	21–27	[76]
Recycled cotton fabric	Chemical (alkali pretreatment, acid hydrolysis) and mechanical processes	Length: 38–424 nm, Diameter: 2–17 nm	49	[77]
Cotton textile waste	Chemical (alkaline pretreatment, acid hydrolysis, chlorine-free bleaching)	Length: 203.7 ± 68.6–1819.3 ± 328.5 nm, Diameter: 16.5 ± 3.6–248.0 ± 130 nm.	63–83	[62]
Cotton linter	Alkaline pretreatment and acid hydrolysis are classified as chemical methods.	Length: 133 nm, Diameter: 10 nm	59–72	[78]
Cotton gin and waste from textile production	Chemical (acid hydrolysis)	Length: 100–300 nm Diameter: <10 nm	50	[79]
Cotton-polyester blend	Chemical (alkaline treatment, acid hydrolysis), mechanical	Length: 40–400 nm, Diameter: 40–100 nm	-	[80]
100% cotton	Acid hydrolysis	Diameter: 50–150 nm Crystallinity: 80%	73–77	[81]
Cotton textile waste	Chemical (alkaline treatment, ozone bleaching, acid hydrolysis), mechanical	Length: 60–220 nm, Diameter: 10–30 nm	-	[63]
Viscose-rayon and nylon yarn	Chemical (acid hydrolysis), mechanical	Diameter: 65.03 ± 10.15 nm	-	[82]
Cotton and polyester blend (65% cotton, 35% polyester)	Chemical treatments (alkaline treatment, acid hydrolysis)	-	56	[4]
Raw and bleached cotton sliver	Chemical (acid hydrolysis) and mechanical processes	Length: 130–300 nm, Diameter: 8–34 nm	78–88	[83]
Cotton pulp fiber	Biological (enzymolysis)	Length: 250–900 Diameter: 30–45	-	[84]
Cotton cloth waste scraps without dye	TEMPO-mediated oxidation	Spherical CNCs Crystallinity index: 81–85% Crystallite size: 5.1–7.8 nm	73	[81]
Waste cotton clothes	Sulfuric acid (H_2_SO_4)_ hydrolysis and three-step oxidation	Rod-shaped structure Aspect ratio: 10.00	90	[67]
Waste cotton clothes	H_2_SO_4_/HCl hydrolysis Ultrasonication	- Length: 38–424 nm - Diameter: 2 to 17 nm - Aspect ratio: 10–32	49	[77]
Waste cotton fabrics with a cellulose content of 94%.	Microcrystalline cellulose production through H_2_SO_4_ hydrolysis and mechanical methods. Stirring and ultrasonication	MCC particle size: 5–400 μm, MCC volume mean diameter: 49 μm SCNC dimension: 5–100 nm SCNC diameter: 35 nm	MCC: 78 SCNC: 22	[85]

## Data Availability

No new data were created or analyzed in this study. Data sharing is not applicable to this article.
